# HDAC9 promotes glioblastoma growth via TAZ-mediated EGFR pathway activation

**DOI:** 10.18632/oncotarget.3223

**Published:** 2015-02-10

**Authors:** Rui Yang, Yanan Wu, Mei Wang, Zhongfeng Sun, Jiahua Zou, Yundong Zhang, Hongjuan Cui

**Affiliations:** ^1^ State Key Laboratory of Silkworm Genome Biology, Southwest University, Chongqing 400715, P.R. China; ^2^ Department of Neurosurgery, Research Institute of Surgery, Daping Hospital, Third Military Medical University, Chongqing 400042, P.R. China

**Keywords:** HDAC9, TAZ, cell cycle, EGFR, Glioblastoma

## Abstract

Histone deacetylase 9 (HDAC9), a member of class II HDACs, regulates a wide variety of normal and abnormal physiological functions. We found that HDAC9 is over-expressed in prognostically poor glioblastoma patients. Knockdown HDAC9 decreased proliferation *in vitro* and tumor formation *in vivo*. HDAC9 accelerated cell cycle in part by potentiating the EGFR signaling pathway. Also, HDAC9 interacted with TAZ, a key downstream effector of Hippo pathway. Knockdown of HDAC9 decreased the expression of TAZ. We found that overexpressed TAZ in HDAC9-knockdown cells abrogated the effects induced by HDAC9 silencing both *in vitro* and *in vivo*. We demonstrated that HDAC9 promotes tumor formation of glioblastoma via TAZ-mediated EGFR pathway activation, and provide the evidence for promising target for the treatment of glioblastoma.

## INTRODUCTION

Glioblastoma (GBM) is the most common and aggressive primary brain tumor, with an extremely poor prognosis and very few therapeutic advances in the last decade. The current standard of care for patients with GBM provides only palliation with a median survival of about 15 months [1, 2]. The genetic, environmental, bacterial virulence, and many other factors have been in affecting the GBM oncogenic process, but the underlying molecular mechanism is poorly understood [3]. Therefore, better defining the pathogenesis of GBM, looking for useful biomarkers, and exploring novel targets for treatment are urgently demanding.

Mutation and aberrant expression of various HDACs have been often observed in human disease, particularly in some cancers, making them important therapeutic targets for many types of malignancies [4, 5]. However, the contributions of specific HDACs to a given cancer type remain incompletely understood. HDAC9, like most class II HDACs, has a conserved histone deacetylase domain, catalyzes the removal of acetyl moieties in the N-terminal tail of histones, and possesses a long regulatory N-terminal domain to interact with tissue-specific transcription factors and co-repressors [6]. The amino-terminal domain contains highly conserved serine residues that are subjected to phosphorylation. Signal-dependent phosphorylation of HDAC9 is a critical event that determines whether it is localized in the cytoplasm or nucleus [7]. High expression of HDAC9 has been reported in cervical cancer [8]. Likewise, upregulated expression of HDAC9 is associated with poor survival in medulloblastoma patients and in childhood acute lymphoblastic leukemia patients [9, 10]. But, the expression and function of HDAC9 in GBM are still unclear.

The frequent amplification of the epidermal growth factor receptor (EGFR) in GBM was firstly reported in 1985 and it has been confirmed in many subsequent studies [11]. According to statistics, the EGFR gene is amplified in 30–40% of GBM patients, and nearly 50% of them highly express the receptor [12]. EGFR is a member of ErbB family, which is activated by phosphorylating the tyrosine kinase moiety [13, 14]. Following EGFR activation, three major intracellular signaling pathways are activated, including the PI3K/AKT kinase pathway, the Ras-ERK cascade, and the STAT3-dependent signaling events [1, 15–17]. EGFR activation drives cell cycle progression, supports differentiation and migration, and inhibits apoptosis. In GBM cells, EGFR activation is believed to promote cancer cell growth and survival [18–20]. Down-regulation EGFR slowed cell migration [21]. Ninov N *et al* reported that activation of EGFR pathway could increase the expression of cyclin E and CDK2 and then accelerate cell cycle progression [22]. Also, HDAC9 is related to regulating the cell cycle progression. So, we conferred that EGFR/AKT/ERK signaling pathway might involved in the progression which was regulated by HDAC9 in GBM.

Transcriptional coactivator with PDZ-binding motif (TAZ) is a transcription cofactor through interacting with several nuclear factors, and plays a central role in the Hippo pathway, which regulates the size and shape of organ development [23]. TAZ was described as a controlling gene, which is very important for muscle, respiratory epithelia and lung differentiation, osteogenesis and adipogenesis, cardiac and limb development [24]. Recently, TAZ has been identified as an oncogene and has an important role in tumorigenicity of many malignancies, including breast cancer, non-small cell lung cancer, gastric cancer, colon cancer and papillary thyroid carcinoma [25–27]. Krishna *et al*. reported that TAZ regulated mesenchymal differentiation and correlated with higher grade and worse overall survival in gliomas [28]. Furthermore, there's evidence to show that HDAC inhibitors (HDIs) could decrease the TAZ expression [29].

In this paper, we investigated the function of HDAC9 in GBM development and progression. Our studies revealed that HDAC9 promotes GBM growth via TAZ-mediated EGFR pathway activation, and provide the evidence for promising target for the treatment of glioblastoma.

## RESULTS

### High expression of HDAC9 correlates with poor patient prognosis

**Figure 1:**
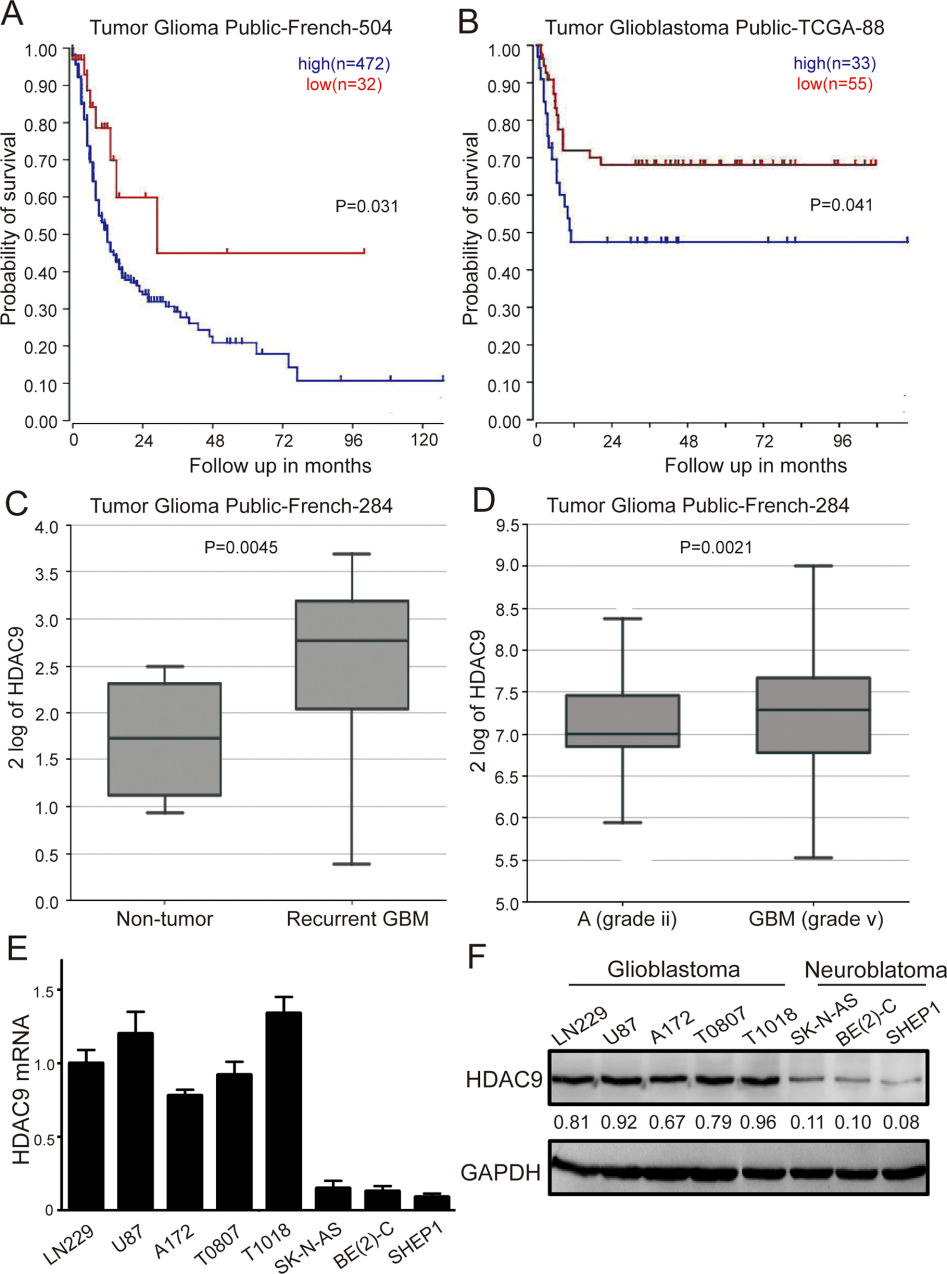
High HDAC9 expression is a prognostic indicator of poor survival in glioblastoma patients **(A)** Kaplan–Meier analysis of progression-free survival for the Frence database with the log rank test *P* value indicated. **(B)** Kaplan–Meier analysis of progression-free survival for the TCGA database with the log rank test *P* value was indicated. **(C)** Box plot of HDAC9 expression levels from non-tumor and recurrent GBM patients was shown. **(D)** Box plot of HDAC9 expression levels in the stage 2 and 5 tumors. **(E)** mRNA level of HDAC9 in glioblastoma cell lines, primary GBM cells and neuroblastoma cell lines by quantitative real time-PCR was analyzed. Values are shown as the mean ± SD **(F)** Western blot assay of HDAC9 expression in GBM cell lines, primary GBM cells and neuroblastoma cell lines was performed; representative blots are shown. Values are shown as the mean ± SD, *p < 0.05, **p < 0.01.

To determine whether alterations at the genetic locus of HDAC9 could be implicated in GBM patient prognosis, survival data from R2 genomics analysis and visualization platform database were used to evaluate the effects of HDAC9 on overall patient survival. HDAC9 was highly expressed in 472 out of 504 cases of glioma, and high expression very significantly correlated with reduced patient survival in French's data, *p* = 0.031 (Figure 1A). Similarly, in TCGA data set consisting of 88 patients, there were 33 cases with upregulation HDAC9, also confirmed that high level of HDAC9 was associated with poor prognosis, *p* = 0.041 (Figure 1B). Furthermore, contrasting to normal tissue and low grade astrocytoma, HDAC9 was significantly upregulated in GBM patients according to French's data, *p* < 0.05 (Figure 1C and 1D). Lastly, we measured the HDAC9 expression of GBM cells by quantitative real time-PCR and western blot assay, and we found that HDAC9 was commonly expressed in GBM cell lines (A172, U-87 MG, LN229) and primary GBM cells from T0807 and T1018 specimen, but it was low expressed in neuroblastoma cell lines (Figure 1E, 1F). All these results indicated that HDAC9 might function as an oncogene involved in the development and progression of GBM.

### HDAC9 is essential for proliferation of GBM cells

**Figure 2:**
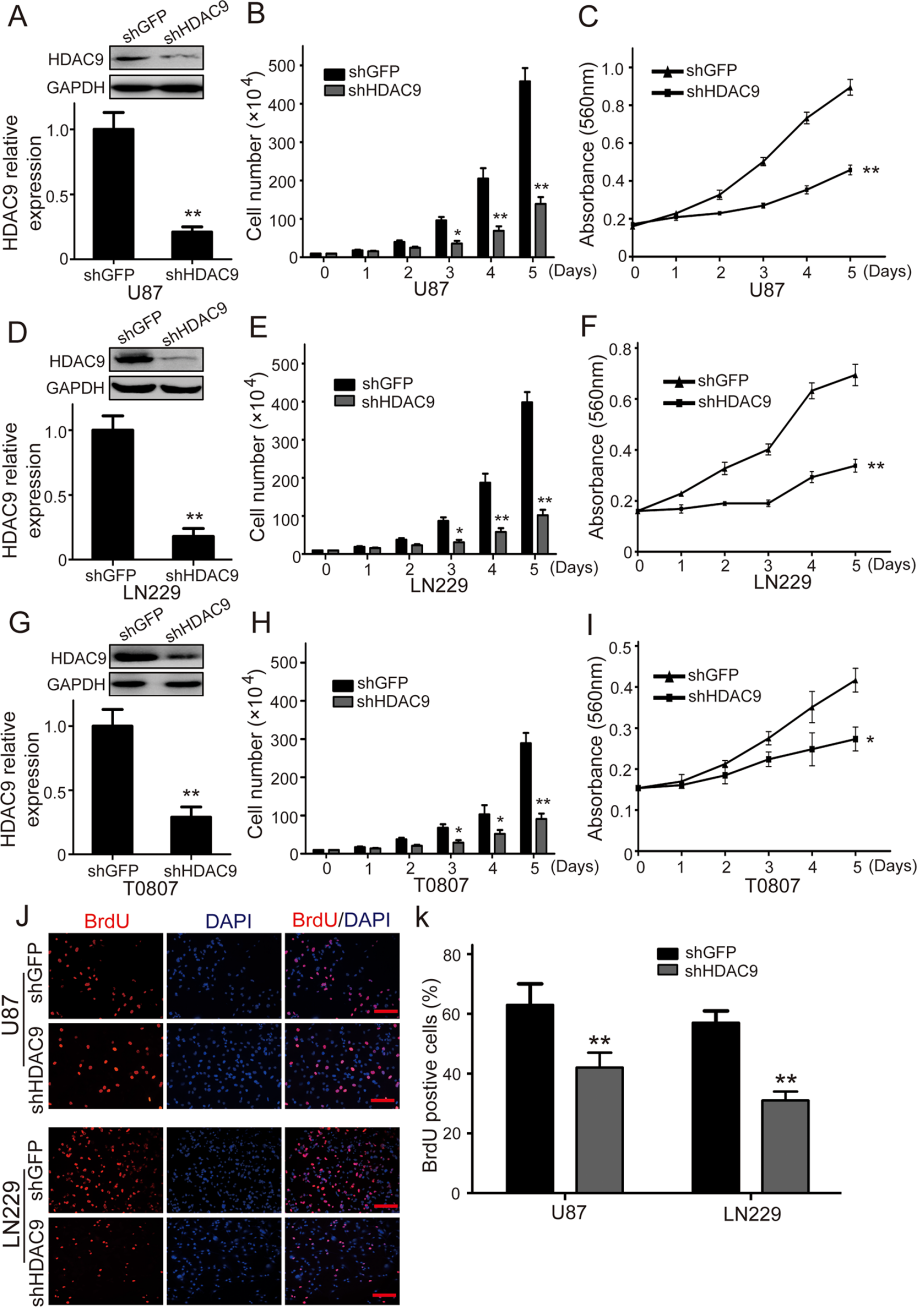
Knockdown of HDAC9 inhibits GBM cell growth and proliferation **(A)** Western blot assay was used to characterize the expression of HDAC9 in HDAC9-knockdown U87 cells. **(B)** The effect of HDAC9 on the proliferation of U87 cells. **(C)** The effect of HDAC9 on the viability of U87 cells. **(D)** Western blot assay was used to characterize the expression of HDAC9 in HDAC9-knockdown LN229 cells. **(E)** The effect of HDAC9 on the proliferation of LN229 cells. **(F)** The effect of HDAC9 on the viability of LN229 cells. **(G)** Western blot assay was used to characterize the expression of HDAC9 in HDAC9-knockdown T0807 cells. **(H)** The effect of HDAC9 on the proliferation of T0807 cells. **(I)** The effect of HDAC9 on the viability of T0807 cells. **(J, K)** Image and quantification of U87 and LN229 cells positive for Brdu staining. All data are shown as the mean ± SD, *p < 0.05, **p < 0.01. All *p* values are based on analysis control versus treatment.

To address the importance of HDAC9 in cell growth and proliferation, we utilized the human glioblastoma cell lines U-87 MG (U87) and LN229, as well as primary cells obtained from GBM patients. Cells were infected with Lentivirus carrying small hairpin RNA (shRNA) constructing against HDAC9 (shHDAC9) or a shGFP control and were subsequently selected by puromycin. Western blot analysis showed that HDAC9 was significantly down-regulated after knockdown by shRNA in different GBM cells (Figure 2A, 2D, 2G). Next, we investigated the proliferation kinetics of GBM cells by using cell growth curve and MTT assay. The growth curve revealed that knockdown of HDAC9 in GBM cells resulted in a significant growth inhibition (Figure 2B and 2H). Furthermore, MTT assay confirmed that down-regulation of HDAC9 induced a significant decrease in cell viability (Figure 2C, 2F and 2I). Above data were confirmed by BrdU incorporation in the U87 and LN229 cell lines, where the HDAC9-knockdown cells showed over a 40% reduction in DNA synthesis compared to control cells in the two cell lines (Figure 2J and 2K). These results demonstrated that HDAC9 was essential for proliferation of GBM cells.

### HDAC9 promotes colony formation *in vitro* and tumor formation of GBM cells *in vivo*

**Figure 3:**
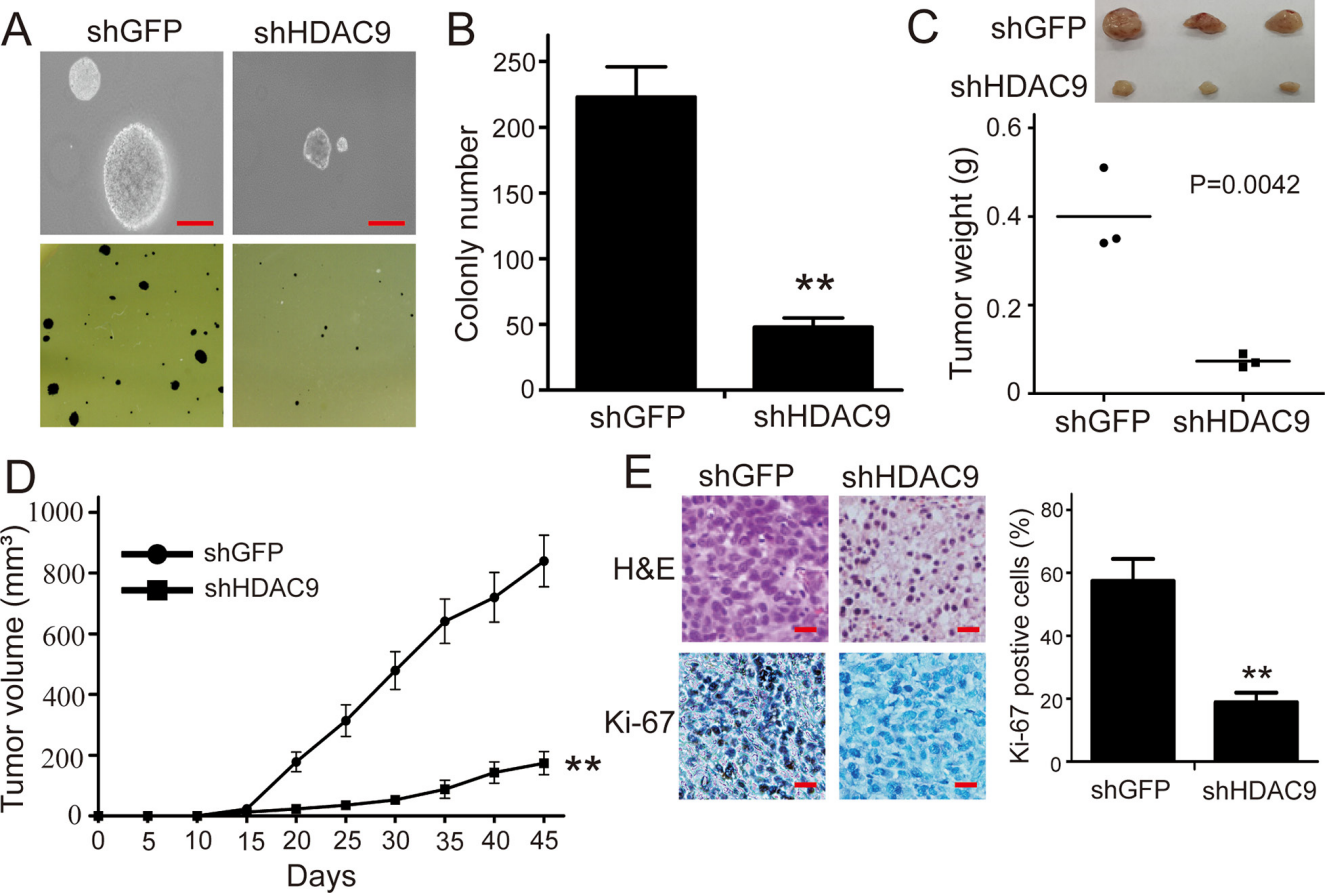
Knockdown of HDAC9 impairs colony formation and tumor formation of U87 cells in immunodeficient mice **(A, B)** The effects of HDAC9 on the colony formation in HDAC9-knockdown U87 cells. **(C)** The size and weight of xenograft tumor formed by the HDAC9-knockdown U87 cells. **(D)** The tumor growth curve of HDAC9-knockdown U87 cells injected into immunodeficient mice. The data were analyzed with 2-tailed Student *t* test, and the *P* value is indicated. **(E)** Immunohistochemical staining for Ki67 in tumor tissues, shHDAC9: shRNA for HDAC9; shGFP: shRNA for control. All data are shown as the mean ± SD, *p < 0.05, **p < 0.01. All *p* values are based on analysis control versus treatment.

To further assess the effects of HDAC9 expression in colony formation, soft agar assay was employed *in vitro*. As shown in Figure 3A and 3B, the colonies were smaller and lesser in HDAC9-knockdown cells compared with the controls (Figure 3A, 3B). Xenograft experiment showed that the tumors formed by the HDAC9-knockdown U87 cells grew much slower (Figure 3D). At the termination of the experiment, the mice were sacrificed, and the tumors were excised; the weight of wet tumors formed by HDAC9-knockdown cells was lower (Figure 3C, *p* = 0.0042). These results indicate that HDAC9 could promote the tumor growth of GBM cells.

To determine whether HDAC9 enhances the tumor progression of GBM cells by promoting cell proliferation, the expression of Ki67, a well-known cell proliferation marker, was examined in the tumor xenografts tissues by IHC staining. As shown in Figure 3E, the expression of Ki67 in the tumor tissues formed by the HDAC9-knockdown U87 cells was decreased compared with the shGFP cells. The results suggested that HDAC9 most likely enhanced the tumor progression of GBM cells by promoting cell proliferation.

### HDAC9 accelerates cell cycle progression in GBM cells

**Figure 4:**
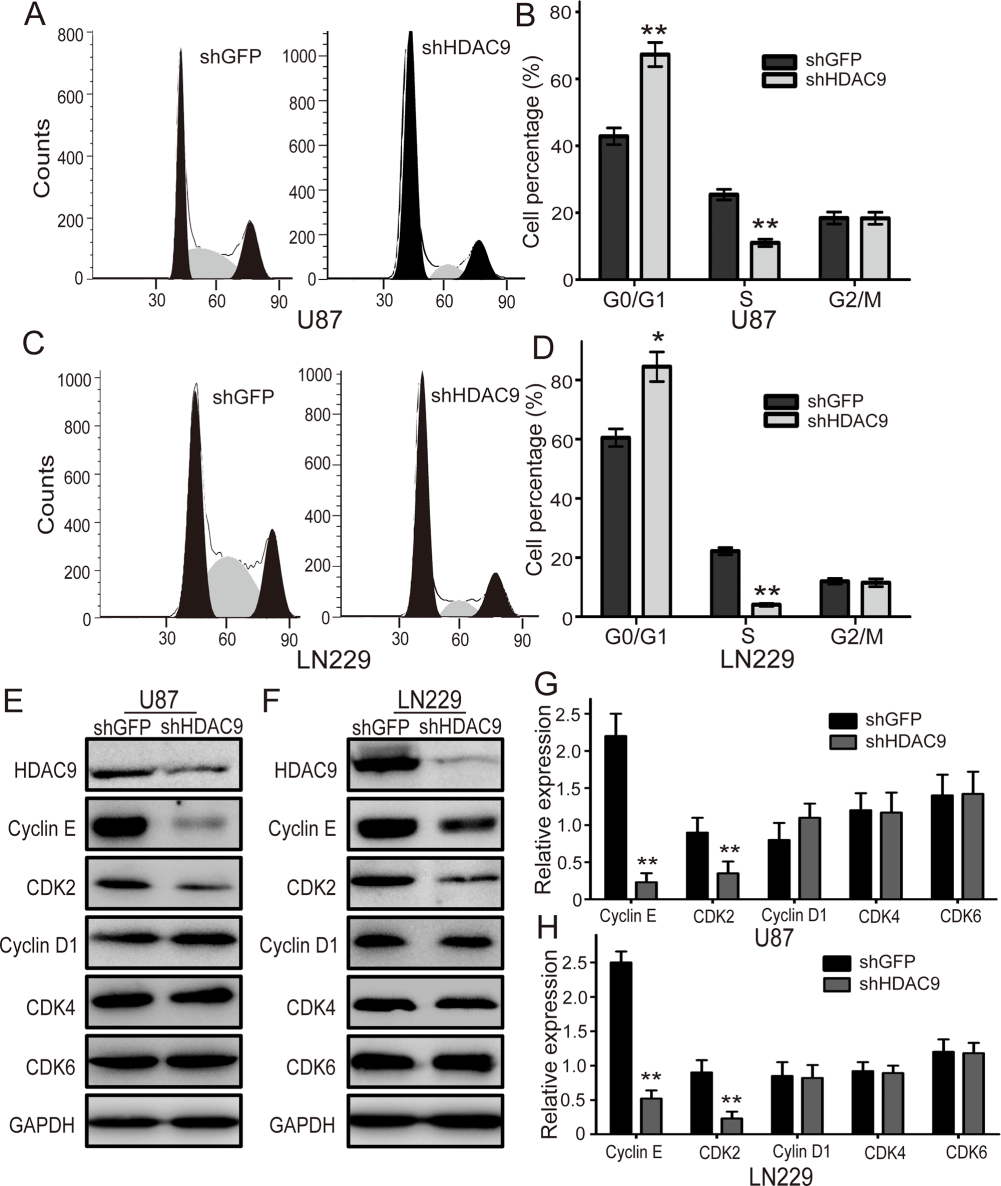
Knockdown of HDAC9 induces cell cycle arrest in G1 phase **(A)** The cell cycle of HDAC9-knockdown U87 cells was analyzed by flow cytometry. **(B)** The effects of HDAC9 on the cell cycle of U87 cells. **(C)** The cell cycle of HDAC9-knockdown LN229 cells was analyzed by flow cytometry. **(D)** The effects of HDAC9 on the cell cycle of LN229 cells. **(E, F)** Western blot analysis of some cyclins and CDKs expression in HDAC9-knockdown cells; representative blots are shown. **(G, H)** Quantitative analysis of cyclins and CDKs expression in HDAC9-knockdown cells; GAPDH was used as a loading control; student's *t*-test was carried out. All data are shown as the mean ± SD, *p < 0.05, **p < 0.01.

Since the cell cycle progression usually regulates cell proliferation, the U87 and LN229 cell cycle was analyzed by flow cytometry to examine whether HDAC9 promotes cell proliferation by accelerating the cell cycle progression. Representative histograms and the results are summarized in Figure 4A, 4B, 4C and 4D, and HDAC9 knockdown resulted in a markedly increase in the percentage of both U87 and LN229 cells in G1 phase. The results demonstrated that knockdown of HDAC9 could induce cell cycle arrest at G1 phase. To confirm the results, we measured the expression of some cyclins and CDKs which could promote cells to pass the G1/S checkpoint. We found that the expression of cyclin E and CDK2 were reduced in the HDAC9-knockdown cells, but no significant changes were found in the expression of cyclin D1, CDK4 and CDK6 (Figure 4E, 4F, 4G and 4H). These results suggested that HDAC9 accelerated cell cycle progression by upregulating of the CCNE-CDK2 complex.

### HDAC9 potentiates the EGFR pathway in GBM

**Figure 5:**
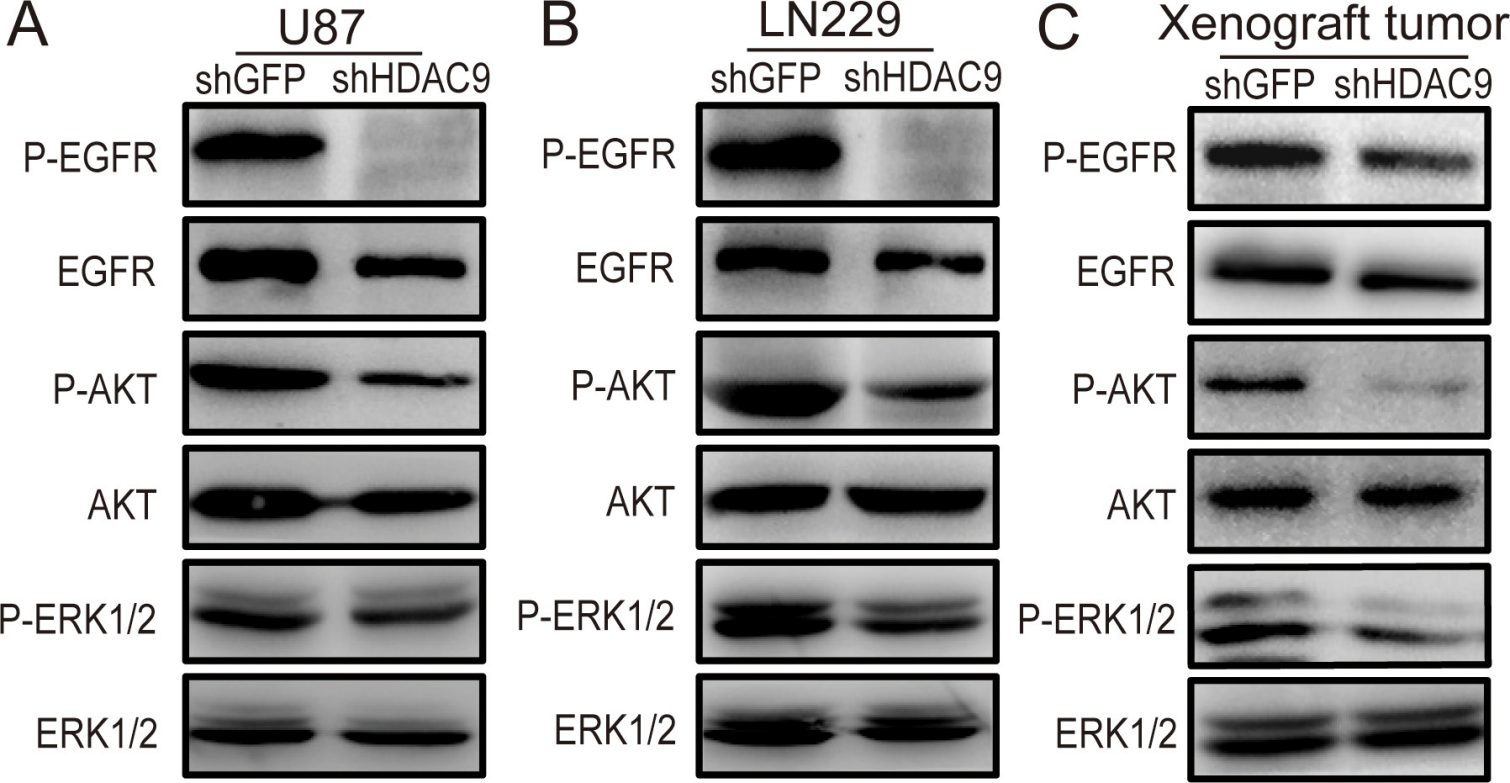
Knockdown of HDAC9 inhibits the activity of the EGFR/AKT/ERK pathway **(A)** The expression of p-EGFR, EGFR, p-AKT, AKT, p-ERK1/2 and ERK1/2 in HDAC9-knockdown U87 cells was measured by western blot assay. **(B)** Representative blots showing the expression of p-EGFR, EGFR, p-AKT, AKT, p-ERK1/2 and ERK1/2 in HDAC9-knockdown LN229 cells. **(C)** The expression of p-EGFR, EGFR, p-AKT, AKT, p-ERK1/2 and ERK1/2 in tumor xenografts was measured by western blot.

Some studies have demonstrated that activation of the epidermal growth factor receptor (EGFR) could accelerate cell cycle progression by activating the cyclin E-CDK2 kinase, and then promote cell proliferation. EGFR is a crucial signaling molecule, and PI3K/AKT and Ras-ERK are important downstream signaling pathways of EGFR. Therefore, the expression of p-EGFR, p-AKT and p-ERK1/2 proteins was measured by western blot assay in HDAC9-knockdown and shGFP GBM cells. Representative blots for U87 and LN229 cells are shown in Figure 5A and 5B. To test whether the expression of HDAC9 is also associated with EGFR signaling *in vivo*, the expression of the p-EGFR, p-AKT and p-ERK1/2 proteins was examined in the xenograft tumor tissues formed by HDAC9-knockdown U87 cells (Figure 5C). The expression of these proteins in HDAC9-knockdown cells and in the tumor tissues formed by the HDAC9-knockdown U87 cells was all markedly reduced compared with their controls. All these results suggested that the HDAC9-promoted proliferation and tumor formation of GBM cells were possibly mediated by potentiating the EGFR signaling pathway.

### HDAC9 interacts with TAZ and enhances the expression of TAZ

It has been reported that HDAC inhibitors (HDIs) could decrease the expression of TAZ and inhibit cell proliferation [29]. However, there is no any report about the relationship of HDAC9 with TAZ.

**Figure 6:**
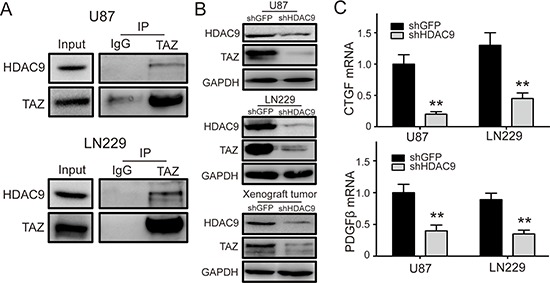
HDAC9 interacts with TAZ and enhances the expression of TAZ **(A)** Co-immunoprecipitation (Co-IP) of HDAC9 and TAZ (U87 and LN229). IgG represents a control antibody used for IPs. Five hundred micrograms of lysates were used for each IP reaction and 50 μg lysates were used as input controls. **(B)** The expression of TAZ in HDAC9-knockdown U87, LN229 cells and xenograft rumors was measured by western blot. **(C)** The mRNA levels of two different TAZ target genes CTGF and PDGFβ in HDAC9-knockdown cells were analyzed by real-time PCR. All data are shown as the mean ± SD, *p < 0.05, **p < 0.01.

We hypothesized that TAZ might be subject to HDAC9 regulation and therefore analyzed binding of HDAC9 to TAZ. We detected HDAC9 after immunoprecipitation of endogenous TAZ from lysates of GBM cells. As shown in Figure 6A, HDAC9 could interact with TAZ. To further examine the effect of HDAC9 activity on TAZ, we measured the protein levels of TAZ in HDAC9-knockdown cells and the xenograft tumors by a western blot assay. The results revealed that the expression of TAZ was reduced in the HDAC9-knockdown cells and the xenograft tumors compared to their controls (Figure 6B). We also analyzed the expression of two different TAZ target genes by quantitative real-time PCR. Knockdown of HDAC9 inhibited induction of CTGF and PDGFβ mRNA expression in GBM cells (Figure 6C). These results demonstrated that HDAC9 interacted with TAZ and enhanced the expression of TAZ.

### TAZ is a crucial downstream effector of HDAC9 in GBM cells

**Figure 7:**
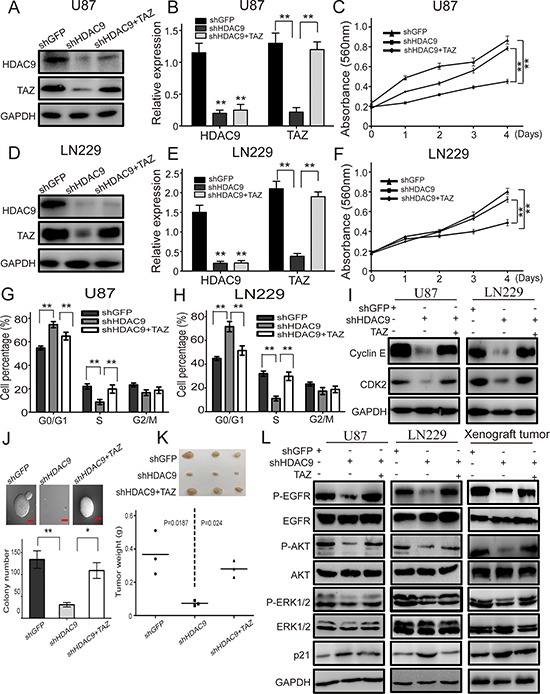
Overexpression of TAZ in HDAC9-knockdown cells abrogates the effects induced by HDAC9 silencing **(A, B)** Western blot assay was used to characterize the expression of HDAC9 and TAZ in the TAZ-overexpressed HDAC9-knockdown U87 cells. **(C)** The effects of TAZ overexpression on the proliferation of HDAC9-knockdown U87 cells. **(D, E)** Western blot assay was used to characterize the expression of HDAC9 and TAZ in the TAZ-overexpressed HDAC9-knockdown LN229 cells. **(F)** The effects of TAZ overexpression on the proliferation of HDAC9-knockdown LN229 cells was measured by MTT assay. **(G, H)** The effects of TAZ overexpression on the cell cycle of HDAC9-knockdown cells. **(I)** The expression of cyclin E and CDK2 in TAZ-rescued HDAC9-knockdown cells were measured by western blot, GAPDH levels were showing as a loading control. **(J)** The effects of TAZ overexpression on colony formation of HDAC9-knockdown U87 cells was analyzed by soft agar assay. **(K)** The effects of TAZ overexpression on tumor formation *in vivo* of HDAC9-knockdown U87 cells. **(L)** Representative blots showing the expression of p-EGFR, EGFR, p-AKT, AKT, p-ERK1/2, ERK1/2 and p21 in the TAZ-overexpressed HDAC9-knockdown cells and xenograft tumor. All data are shown as the mean ± SD, *p < 0.05, **p < 0.01.

**Figure 8:**
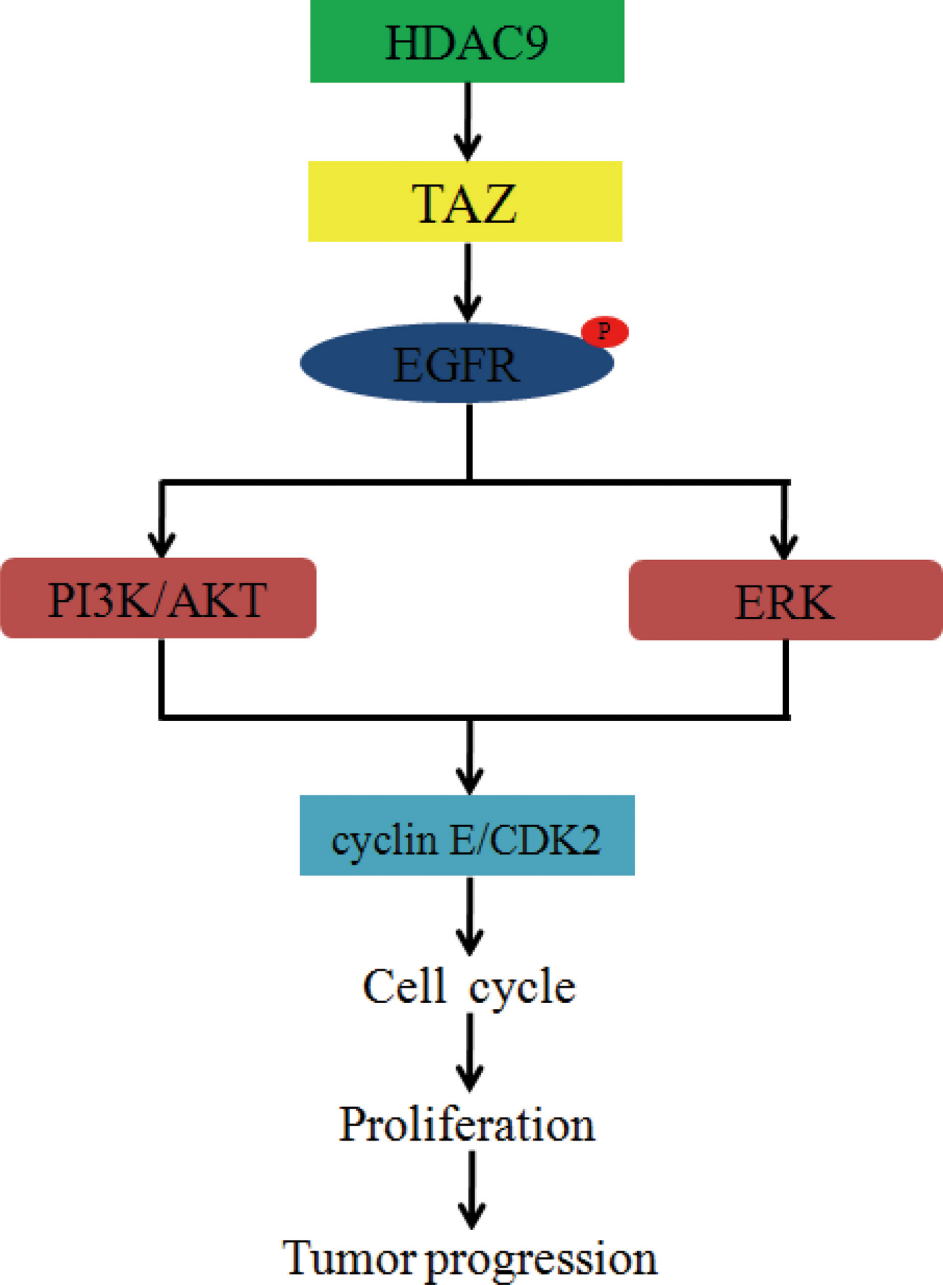
Diagram for mechanism by which HDAC9 promotes cell proliferation and tumor progression in glioblastoma HDAC9 interacts with TAZ, enhances the expression of TAZ, and then phosphorylates EGFR. The phosphorylation of EGFR activates PI3K and ERK signaling pathways, increases the expression of cyclin E and CDK2, accelerates cell cycle progression, and then promotes cell proliferation and tumor progression in GBM.

As HDAC9 interacted with TAZ and knockdown of HDAC9 reduced the expression of TAZ, we hypothesized that TAZ might be a downstream effector of HDAC9 in GBM cells. To prove the inference, we forcedly expressed TAZ in HDAC9-knockdown cells, which induced TAZ down regulation. Western blot assay suggested that the expression of TAZ was rescued in the HDAC9-knockdown cells (Figure 7A, 7B, 7D and 7E). MTT assay was employed to analyze the abilities of cell proliferation and growth; results revealed that the proliferation ability was rescued after TAZ overexpressed in HDAC9-knockdown cells (Figure 7C and 7F). Cell cycle was analyzed by flow cytometry to examine whether TAZ promoted HDAC9-knockdown cells proliferation by rescuing the cell cycle progression. Overexpressed TAZ in HDAC9-knockdown cells significantly abrogated the effects of HDAC9 knockdown on the cell cycle progression. Similarly, the expression of cyclin E and CDK2 were also upregulated in the TAZ-rescued HDAC9-knockdown cells (Figure 7G–7I). The results were confirmed by soft agar assay in U87 cells, where the ability of colony formation was rescued after TAZ overexpressed in the HDAC9-knockdown cells (Figure 7J). The xenograft experiment revealed that the ability of tumor formation of TAZ-overexpressed HDAC9-knockdown U87 cells *in vivo* was also rescued (Figure 7K). Then we measured the activation of EGFR and its downstream signaling, the phosphorylation levels of EGFR, AKT and ERK1/2 were increased in the TAZ-rescued HDAC9-knockdown cells and xenograft tumors. The tumor suppressor p21 expression was decreased in the TAZ-overexpressed shHDAC9 cells and xenograft tumors (Figure 7L). Taken together, all the results demonstrated that TAZ was a crucial downstream effector of HDAC9 and HDAC9 promoted cell proliferation and tumor formation via TAZ-mediated EGFR activation in GBM Figure 8.

## DISCUSSION

HDAC9, a member of class II HDACs, regulates a wide variety of normal and abnormal physiological functions, including T-regulatory cell function, cardiac growth, neuronal disorders, and muscle differention [30–32]. Recent studies have observed that HDAC9 plays an important role in tumorigenesis and exerts a dual role in different cancers, including cervical cancer, medulloblastoma and acute lymphoblastic leukemia [9, 10]. However, to our knowledge, the role of HDAC9 in glioblastoma remains unclear. We first evaluated the clinical significance of HDAC9 in a very large cohort of GBM patients and found a significant positive association of HDAC9 expression with overall survival. Furthermore, we found that HDAC9 was commonly expressed in GBM cells, suggesting that HDAC9 may promote the development and progression of GBM.

Subsequently, through shRNA knockdown, the HDAC9 expression was found to be positively related to the proliferation of GBM cells. Tumor xenograft experiments in SCID mice indicated that HDAC9 significantly promoted tumor growth *in vivo*. Moreover, immunostaining assays revealed that the tumor tissues formed by HDAC9-knockdown cells had much weak Ki67 expression, suggesting that HDAC9 promoted the tumor formation of glioblastoma cells by accelerating cell proliferation. Furthermore, a cell cycle analysis by FACS revealed that decreased HDAC9 expression induced cell cycle arrest at G1 phase. All of our findings together indicated that HDAC9 promotes the development and progression of GBM.

Recently, EGFR was found to be highly expressed in GBM cells, the activation of EGFR could accelerate cell cycle progression and promote cell proliferation by activating its downstream signaling pathway (PI3K/AKT and Ras-ERK) [13, 17, 18]. In this study, we found that knockdown of HDAC9 suppressed EGFR and its downstream signaling pathways activation in GBM cells and xenograft tumors. These results suggested that HDAC9 potentiates the EGFR/AKT/ERK pathway, and then accelerates cell cycle and promotes proliferation in GBM cells.

An increasing number of studies have demonstrated that TAZ enhanced the activity of EGFR, accelerated cell proliferation and promoted tumorigenesis in many types of malignancies [33]. Furthermore, a previous research reported that HDIs could decrease the expression of the Hippo transducer TAZ and suppressed cell growth and proliferation [29, 34]. However, the relationship between HDAC9 and TAZ is unclear. In the present study, we found that HDAC9 interacted with TAZ and enhanced TAZ expression, suggested that TAZ might be a downstream effector of HDAC9. To confirm this consequence, we overexpressed the TAZ in the HDAC9-knockdown cells, and found that all the effects induced by HDAC9 silencing were abrogated. These results demonstrated that TAZ is a crucial downstream effector of HDAC9 in GBM cells. B Mao *et al* reported that the histone deacetylase SIRT1 could regulate the acetylation of YAP (a homologous gene of TAZ) and regulated YAP expression and location [34]. We speculated that HDAC9 might up-regulates TAZ expression via regulating TAZ acetylation or indirect mechanism such as via regulating acetylation of histones. As TAZ is a Hippo transducer, and the expression of two Hippo pathway target genes CTGF and PDGFβ was decreased after HDAC9 silencing, suggested that HDAC9 might promote TAZ expression by regulating some upstream genes of Hippo pathway [35]. Therefore, further investigation is necessary to clarify the mechanism that HDAC9 up-regulated TAZ expression and promoted progression of glioblastoma.

In summary, for the first time we demonstrated that HDAC9, as a novel oncogene, promotes GBM growth via TAZ-mediated EGFR pathway activation. Our studies indicated that HDAC9 could be a novel and promising target for GBM treatment.

## MATERIALS AND METHODS

### Cell lines, primary cells and cell culture

Human glioblastoma cell lines (U87, LN229, and A172) were purchased from American Type Culture Collection (ATCC, Rockville, MD, USA). Primary glioblastoma cells (T0806, T1018) were obtain from Daping Hospital, Third Military Medical University (Chongqing, China) and written consent was obtained from patients prior to experiment. All the cells were cultured in a 1:1 mixture of Dulbecco's Modified Eagle's Medium and Ham's nutrient mixture F12 (DMEM/F-12, Life Technologies, Grand Island, NY, USA), and carried out at 37°C with 5% CO_2_. All media were supplemented with 10% fetal bovine serum (FBS, Life Technologies, Grand Island, NY, USA).

### Vector construction and infection

The small interfering RNA expression vector that expresses HDAC9-specifi short hairpin RNA (shHDAC9), TAZ-specific short hairpin RNA (shTAZ) and GFP-specific short hairpin RNA (shGFP) were purchased from GenePharma Co., Ltd (Shanghai, China). Human full-length TAZ cDNA was obtain from Queen's University Richardon Lab, the DNA fragment was subsequently cloned into pCDH-CMV-MCS-EF1-copGFP vector to generate the recombinant plasmid [35]. The HDAC9 shRNA, TAZ shRNA and TAZ overexpression vectors were transfected into 293FT cells using the Lipofectamine 2000 reagent (Invitrogen, Carlsbad, CA, USA) according to the manufacturer's protocol, and then the Lentivirus were infected into GBM cells. The transfected cells were selected with puromysin for 1 week, and drug-resistant cells were collected, expanded and identified.

### Cell proliferation and viability assays

Cells were seeded and cultured in 6-well plates at the concentration of 1 × 10^5^ cells/well. The cells were harvested and counted daily for 5 days using a hemocytometer, and cell proliferation was assessed by cell growth curves. To test cell viability, 1 × 10^3^ cells were cultured in 96-well plates for 5 days, and MTT assay was performed according to the manufacturer's protocol. All experiments were performed independently in triplicate.

### BrdU staining

For BrdU immunofluorescent staining, cells were grown on coverslips, and incubated with 10 μg /ml BrdU (Sigma) for 30 min, then washed with phosphate buffered saline (PBS) and fixed in 4% paraformaldehyde (PFA) for 20 min. Subsequently, cells were pre-treated with 1 mol/L HCl, and blocked with 10% goat serum for 1 h, followed by a monoclonal rat primary antibody against BrdU (1:200, ab6326, Abcam, Cambridge, MA, USA) for 1 h and Alexa FluorR^®^ 594 goat anti-rat IgG secondary antibody, (H+L; Invitrogen). DAPI (300 nM) was used for nuclear staining; the percentage of BrdU was calculated at least from 10 microscopic fields (Nikon 80i, Nikon Corporation, Tokyo, Japan).

### Soft agar assay

In total, 2 × 10^3^ cells were mixed with 0.3% Noble agar in growth medium and plated into six-well plates containing a solidified bottom layer (0.6% Noble agar in growth medium). The colonies were photographed after 14 to 21 days and recorded.

### Tumor xenograft experiment

Female SCID mice (4 weeks old) were purchased and housed in an SPF room that was maintained at a constant temperature (22°C–25°C) and humidity (40%–50%). Tumor cells (1 × 10^6^) were injected into the subcutis on the dorsum of each mouse. The tumor size was measured using a vernier caliper every week, and the volume was calculated with the following formula: V = (length × width^2^)/2. At the termination of the experiment, the tumor mass was harvested, weighed, and stored for immunostaining or protein extract. All studies were approved by the Animal Care and Use Committee of Southwest University.

### Flow cytometry

For cell cycle analysis, 1 × 10^6^ cells were harvested and washed twice with cold PBS, followed by fixation with ice-cold 70% ethanol overnight at 4°C. After washing twice with PBS, the cells were incubated with propidium iodide (PI) (BD Biosciences, San Jose, CA, USA) and RNaseA for 30 min at room temperature. The cells were then analyzed using a FACS C6 (BD Biosciences, San Jose, CA, USA) with CellQuest software.

### Quantitative real-time PCR (qRT-PCR)

Total RNA was extracted using TRIzol (Invitrogen) according to the manufacturer's protocol and RNA was reverse transcribed into cDNA using M-MLV reverse transcriptase (Promega Corporation, Madison, WI, USA). The HDAC9, CTGF and PDGFβ mRNA transcripts were determined using the SYBER Green PCR Master mix (Takara Bio, Inc., Shiga, Japan) by quantitative RT-PCR. RT-qPCR reactions in triplicate were conducted using the OneStep plus7500 real-time PCR system (Bio-Rad). The individual values were normalized to that of the GAPDH control.

### Western blotting and co-immunoprecipitation

The lysates from cells and fresh tissues were separated by SDS-PAGE and transferred onto PVDF membranes (Millipore, USA). After blocking with 5% BSA, the membranes were incubated with a primary antibody against human HDAC9 (1:1000, Abcam), GAPDH (1:1000, Beyotime), CDK2 (1:500, Santa Cruz), CDK4 (1:500, Santa Cruz), CDK6 (1:500, Santa Cruz), Cyclin D1 (1:500, Santa Cruz), Cyclin E (1:500, Abcam), EGFR (1:2000, Cell Signaling), phospho-EGFR (Tyr1068) (1:1000, Cell signaling), AKT (1:500, Cell Signaling), phospho-AKT (Ser473) (1:1000, Cell signaling), ERK1/2 (1:500, Cell Signaling), phospho-ERK1/2 (Thr202/Tyr204) (1:2000, Cell Signaling), TAZ (1:200, Santa Cruz) or p21 (1:500, Cell Signaling) at 4°C overnight, followed by appropriate HRP-conjugated secondary antibodies. Immunocomplexes were visualized by ECL Western blot analysis detection system. For co-immunoprecipitation (co-IP) studies, cells were grown in 10-cm-diameter cell culture dishes. Cells were lysed and the resulting supernatants were incubated on a rocker with 2 μg TAZ antibody for 1 h and then with 20 μl protein A/G PLUS Agarose (Santa Cruz) overnight at 4°C. The immunoprecipitates were collected and then subjected to SDS/PAGE analysis.

### Immunohistochemistry staining

Paraffin embedded tumor tissues were sectioned at 5 μm, deparaffinized, and rehydrated. For antigen retrieval, sections were treated for 20 minutes at 95°C in 10 mmol/L citrate buffer (pH 6.0) in a laboratory microwave oven and subsequently washed in PBS. For immunohistochemistry, after quenching of endogenous peroxidase activity and blocking with normal goat serum, sections were incubated sequentially with Ki67 primary antibodies (1:100, clone 550609, BD pharmingen), biotinylated goat anti-mouse IgG, and the ABC reagent (Vector Laboratories). The immunostaining was visualized with 3, 3′-diaminobenzidine (Sigma). Sections were then counterstained with hematoxylin before being examined using a light microscope.

### Patient data analysis

Patient data and gene expression datasets were obtained from R2: microarray analysis and visualization platform (http://hgserver1.amc.nl/cgi-bin/r2/main.cgi). All prognosis analyses were conducted online, and all data and *P* values (log-rank test) were downloaded. Kaplan–Meier analysis and the resulting survival curves were performed using GraphPad Prism (version 6.0). All cutoff values for separating high and low expression groups were determined by the online R2 database algorithm.

### Statistical analysis

All observations were confirmed by at least three independent experiments. Quantitative data are expressed as the mean ± standard deviation. Two-tailed Student's *t*-test was performed for paired samples. *P* < 0.05 was considered statistically significant.
